# Evaluation of cadmium and mercury on cardiovascular and neurological systems: Effects on humans and fish

**DOI:** 10.1016/j.toxrep.2023.04.009

**Published:** 2023-04-18

**Authors:** Azza Naija, Huseyin Cagatay Yalcin

**Affiliations:** Biomedical Research Center, Qatar University, Doha, Qatar

**Keywords:** Cadmium, Mercury, Fish, Humans, Heart, Brain

## Abstract

Chemicals are at the top of public health concerns and metals have received much attention in terms of toxicological studies. Cadmium (Cd) and mercury (Hg) are among the most toxic heavy metals and are widely distributed in the environment. They are considered important factors involved in several organ disturbances. Heart and brain tissues are not among the first exposure sites to Cd and Hg but they are directly affected and may manifest intoxication reactions leading to death. Many cases of human intoxication with Cd and Hg showed that these metals have potential cardiotoxic and neurotoxic effects. Human exposure to heavy metals is through fish consumption which is considered as an excellent source of human nutrients. In the current review, we will summarize the most known cases of human intoxication with Cd and Hg, highlight their toxic effects on fish, and investigate the common signal pathways of both Cd and Hg to affect heart and brain tissues. Also, we will present the most common biomarkers used in the assessment of cardiotoxicity and neurotoxicity using Zebrafish model.

## Graphical Abstract

**Figure:**
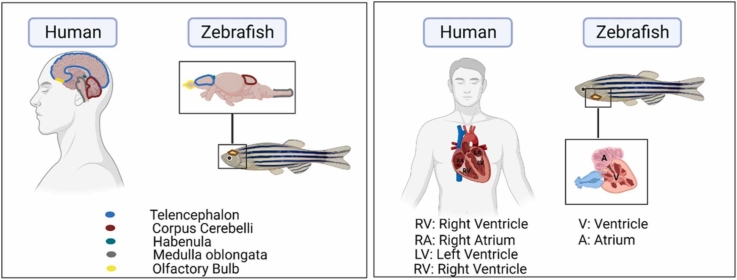

## 1 Introduction

For a long time, humans have coveted metals. Consumed largely for utilitarian purposes, human activities have brought no change to heavy metal (HM) volumes. There is neither creation nor deletion; metals have only been changed in terms of concentrations, speciation, and distributions through new modes of dispersal. HMs include toxic metals such as arsenic (As), cadmium (Cd), lead (Pb), and mercury (Hg), and essential trace metals like chromium (Cr), cobalt (Co), copper (Cu), magnesium (Mg), molybdenum (Mo), nickel (Ni), selenium (Se), tungsten (W), vanadium (V), and zinc (Zn). Toxic metals have no useful biological roles in living organisms and HMs remain the most problematic issue threatening human health [1], [2], [3], [4], [5]. The National Poisoning Data System (NPDS) of the American Association of Poison Control Center (AAPCC) reported 8039 cases of single exposures to HMs in 2019 [6]. In its report, the Word Health Organization (WHO) has listed As, Pb, Cd, and Hg among the 10 chemicals of public health concern [7]. In 1993, the International Agency for Research on Cancer (IARC) had classified Cd as a human carcinogen of Group I since it increased lung cancer risk among exposed workers. Cd and Hg have a high affinity to sulfur, which gives them the characteristics of bioaccumulation and toxicity [8], [9].

**Fig. 1:**
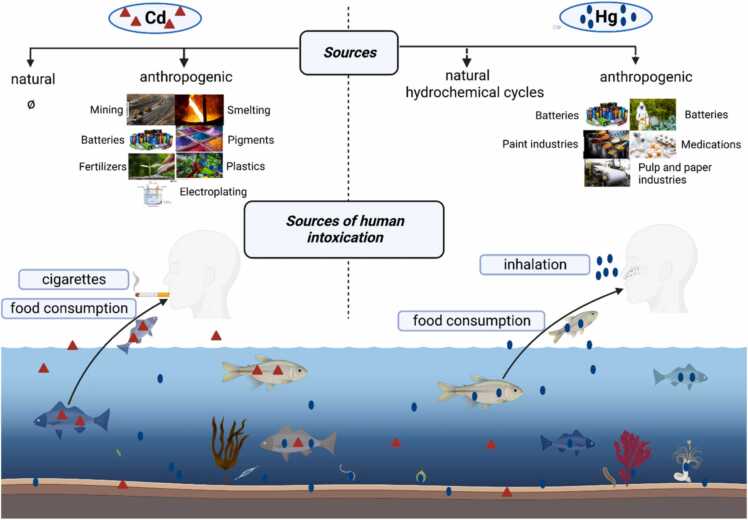
Summary of principle sources of Cd and Hg production and human intoxication leading to cardiotoxicity and neurotoxicity.

Cd toxicity makes it one of the most problematic metals in terms of environmental health [10]. It is principally obtained from activities such as mining, smelting, electroplating, and the production and use of batteries, pigments, fertilizers, and plastics [11]. Cd has a long half-life which was estimated in rats between 200 and 700 days while, in humans, it may exceed 30 years [7]. For Hg, anthropogenic emissions of the metal are estimated at thousands of tons every year, Hg is present in the normal hydro-geochemical cycles and released by natural evaporation to the sea, land surfaces, and volcanos. Hg is mainly used for battery production, paint industry, pesticide fabrication, pulp and paper manufacturers, and medicines (Fig. 1). According to Abadin et al. (1997), absorption of inorganic Hg in humans occurs in the range of 2–38% [12]. In the human body, both Cd and Hg compete for common transport and cellular sites [13], [14] and are able to induce or inhibit enzyme expressions and functions [15], [16].

Heart and brain tissues are not considered as sites of primary exposure as lung, gut, and skin but evidence increasingly indicates that human exposure to Cd and Hg is linked to cardiovascular and neurodegenerative risks [17], [18], [19], [20]. According to the WHO, environmental degradation and HM exposures are responsible for 13–17% of cardiovascular diseases (CVDs) [7] (Fig. 1). These percentages make HMs the most pressing nemeses of human health worldwide. In 1996, Murray and Lopez (1996) followed the progression of CVDs causing death and estimated an increase of 7.4% from 1990 to 2020 [21]**;** but in the last update of WHO, the proportion of CVDs causing death was almost doubled within these 3 decades [22]. Following epidemiological studies, Fagerberg et al. (2015) demonstrated that Cd exposure significantly increased stroke and heart failure [23]. Likewise, Cd levels positively correlated with a 10-year risk of CVDs [24]. Many other studies reported that exposure to Cd is an increasing factor of CVD risks, atherosclerosis, stroke, high blood pressure, and hypertension [25], [26], [27], [28]. Hg was also considered an important risk factor for CVDs. Clinically, Hg exposure consequences include hypertension [29], coronary dysfunction [30], myocardial infarction [31], cardiac arrhythmias, and atherosclerosis [32]. Data presented by Halbach (1990) displayed a strong positive correlation between Hg exposure and increased arterial blood pressure (BP) [33]. Hg is also linked to Reduced Heart Variability (RHV), which may lead to sudden heart arrest (Valera et al., 2008).

Hg is a potential neurotoxic agent. The metal may lead to neurological disorders if we take the examples of studies carried out on the Minamata [34], Faroe Island [35], Amazon basin [33], Finnish [36], and Iraq [17] populations following acute exposures to Hg. On the other hand, **Paquin et al. (2002)** associated high blood Cd levels with motor neurons and sporadic motor diseases [37]. Likewise, children of 7–16 years with high levels of Cd in their bodies displayed a high frequency of attention and social problems as reported by Wang and Rainbow (2007) who also demonstrated that Cd plays a key role in the progression of Parkinson’s and Alzheimer’s diseases [38].

Human exposure to HMs is mainly through other animals. While, as OMEGA-3-rich species, fish are a model of healthy food as it is considered an excellent source of proteins and vitamins (Vit D). It was increasingly recognized that fish consumption prevents CVD-related mortality [39]. Human exposures to Cd and Hg occur mainly through seafood consumption which may invalidate the concept of “FISH IS A HEALTHY FOOD”. Both metals easily find their way to water sources where they directly enter fish bodies through water, sediment, and food/prey. Rest is easy where these metals find their way to human systems.

This review highlights the cardiotoxic and neurotoxic effects of HMs, particularly Cd and Hg. In the first and second sections (section I and II) of the present review, we aim to highlight the toxic effects of Cd and Hg on the cardiovascular and neurological systems of humans and fish respectively. In the third section (section III), we will investigate the possible signal pathways of Cd and Hg to affect heart and brain tissues. In the last section (section IV), we will present the different biomarkers used in the assessment of cardiotoxicity and neurotoxicity caused by Cd and Hg in zebrafish species, as an animal model of environmental pollution.

## 2 Section I: effects of Cd and Hg on humans

Exposure to Cd and Hg for extended durations mainly affects cardiovascular and neurological systems. According to Falnoga et al. (2000), the most affected organs are the endocrine glands and kidney cortex for Hg, and kidney cortex and thyroids for Cd intoxications [40].

### 2.1 Chemical characteristics of Cd and Hg

Cd is a soft, malleable, and ductile divalent metal with an atomic number of 48 and a weight of 112.14 u. Considered a bulk metal, Cd is not soluble in water, is not flammable, and its powdered form may release toxic fumes. Cd is a stable metal similar to Hg (atomic number 80, weight 200.59 u) which is the only metallic element known to be liquid at room temperature.

### 2.2 Cardiotoxic impact

Cardiotoxicity is a condition when there are damages or disorders affecting the heart tissue. As a result, the heart may not be able to pump blood throughout the body in a proper way. As mentioned above, up to 17% of CVDs are related to HM exposures. Since the beginning of the industrial development and unlike Hg, we had not witnessed incidents related to Cd. Most cases of human intoxication by Cd come from smoking cigarettes, which is the most important source of Cd exposure [41]. It is estimated that 20 cigarettes release about 30 μg of Cd of which 2–4 μg are inhaled [4]. For the non-smoking population, food is the main source of Cd exposure. Cd can cause vascular tissue damages and promote atherosclerosis [42]. Grandjean and colleagues (2004) studied the incidence of hypertension in young adults exposed to Cd and associated the disease with an increased risk of CVDs [43]. Many other studies also suggested a high positive correlation between Cd levels and hypertension [5], [28], [44], [45]. Added to hypertension, Cd levels may lead to Peripheral Arterial Disease (PAD) and Coronary Heart Disease (CHD) [46].

Aside from its application for many purposes such as dental amalgams, production of chlorine and caustic soda, laboratory uses, niche uses, and firearms, Hg contamination in beauty and infant products has been reported [47]. Hg is also a component of the thimerosal-containing vaccine used as a preservative in pediatric vaccines. Similar to Cd, increased Hg exposure was also suspected to induce hypertension [48], [49] (. Many cases of human intoxication by Hg were reported and revealed a high positive correlation between Hg levels and CVDs, especially through the increase of BP [36], [50], [51], [52]. In a prospective cohort study of 7 years-old Faroese children, methylmercury (MeHg) levels were associated with high BP and a decrease in Heart Rate Variability (HRV) [43], [53]. Another study by Choi et al. (2009) of Faroese whaling men presenting toenail and hair Hg levels displayed an increased carotid Intima-Media Thickness (IMT) and hypertension [49]. These studies and evidence from other studies suggested that people with high Hg levels in urine, blood, hair, and toenail are exposed to high CVD risks [32], [36], [54], [55]. Myocardial Infarction (MI) is also considered a contributing factor to CVDs. Most acute MIs are generated from the blockage of a coronary artery through atherosclerosis which is able to directly limit blood flow in these arteries and generate blood clots leading to a blockage [56], [57]. In a European multicenter study, exposure to MeHg was associated with a higher risk of MI [32]. Salonen et al. (1995, 2000) also displayed that Hg content in hair and fish intake in Eastern Finnish men was positively associated with elevated MI risk and death from CVDs [31], [36]. Accelerated progression of carotid atherosclerosis is another aspect found in this population. In Japan in 1950, the Minamata population has been severely poisoned by MeHg from fish consumption containing Hg discharged to the surrounding sea. HMs were recognized as causal events in atherosclerosis development [58]. Data presented by Yoshizawa et al. (2002) also displayed that Hg exposure was associated with the risk of development and progression of CVDs including atherosclerosis [59].

### 2.3 Neurotoxic impact

Neurotoxicity is defined as any adverse effect on the central (brain and spinal cord) or peripheral (nerves and ganglia outside CNS) nervous system caused by any toxic agent. The neurotoxic risk of Cd for humans in the occupational setting has received little attention in the last 3 decades despite the fact that the metal is considered a putative neurotoxic agent that severely affects CNS with symptoms including headache, vertigo, vision disorders, slowing of vasomotor functioning, peripheral neuropathy, decreased equilibrium and ability to concentrate [60], [61]. Vorobjeva (1957) described the neurotoxic effects of Cd exposure in 160 workers and reported tremors, sweating, dermographia, increased-knee joint reflexes, altered neuromuscular conduction, and optic and sensory disorders [62]. Likewise, Cotter (1958) published a case study of a chemist working with fine-powdered Cd [63]. The author observed an irritable character being manifested after chronic exposure. Other investigators associated Cd levels in the hair of 31 disabled children with a decrease in intelligence and school achievement scores [64].

Many studied examples of the effects of Hg exposure highlighted and supported the nomination of Hg as the most neurotoxic agent. Exposure to high levels of Hg can lead to extensive neurological damages and mortalities [64]. For chronic exposures, neurological damages (visual field conduction, cerebral palsy, deafness), neuromotor (ataxia, muscle weakness, numb limbs, chewing, tremors, spasticity), and neurobehavioral disorders (change in personality, restlessness, anxiety, sleep disturbance, and depression) are well documented. In the Minamata population, a study of 9 adult patients who suffered from prenatal MeHg poisoning was documented. The result displayed lesions in the cerebral cortex and cerebellum [65] and symptoms included dysarthria, constriction of the visual fields, hearing impairment, and sensory disturbances [66]. In Iraq, two cases of human poisoning with Hg were reported for the periods of 1956–1960 and 1971–1972 due to the consumption of seeds treated with fungicides containing high levels of MeHg. People manifested neuropathological symptoms after the two first weeks of bread consumption including paresthria, ataxia, dysarthria, and deafness. Cases of death were also reported when Hg-hair concentrations exceeded 900 pm [2], [3]. Another case study of Hg poisoning occurred in Faroe Island where cases of children exposed to Hg in the prenatal period were reported. The disease displayed many disorders related to attention, memory, language, and motor functions [35].

## 3 Section II: effects of Cd and Hg on fish

The use of fish in cardiotoxicity and neurotoxicity assessments has become essential not only due to their wild consumption by humans but because of their highly similar responses to humans at different intoxication levels.

### 3.1 Cardiotoxic impact

Like humans, the heart is the first organ to become developed and functional in fish. From early blood circulation, start developmental processes of other organs. Despite a few anatomical (2-chambered heart instead of 4 for humans) and circulatory (a single systemic circulation instead of a double circulation for humans) differences, heart failure in fish can manifest similar disorders to humans [67]. There is a shred of growing evidence that both fish protein and oil have several virtues for human health [68]. Fish is an excellent source of nutrients that prevent the occurrence of CVDs especially CVD-related mortality [39]. As mentioned above, cases of human poisoning with Hg are caused by food consumption including fish [69]. Here, the adverse effects of fish-containing toxicants will be more impacted than fish consumption benefits, especially in communities where fish is vital and constitute an important commodity [70]. Once discharged into the environment, most toxic metals find their way into water sources where they are extracted by microorganisms and bioaccumulated in the aquatic food chain. Based on the bioaccumulation and biomagnification processes, high metal levels are found in fish tissues [17], [71]. Many studies investigated the presence of HMs in commercial fish and detected high levels of Cd and Hg which made the fish unsafe for human consumption [72], [73], [74]. Bioaccumulation of metals in fish can occur through three main mechanisms: respiration, adsorption, and ingestion [75]. Metals display different affinities to fish tissues. They are mainly accumulated in the gills, liver, and kidneys [16], [76].

In the cardiac tissue of fish, Cd^2+^ may induce cardiac arrhythmias. Haverinen et al. (2021) showed that Cd^2+^ changed the shape of ventricular action potentials (APs) of rainbow trout hearts [77]. Many studies on zebrafish, Japanese medaka, and rainbow trout displayed that exposure to Cd^2+^ altered heart rates (HRs) and caused changes in the electrocardiogram [78], [79]. In zebrafish larvae, it was found that the baseline HR increased in Cd-treated groups [79]. The authors also noted the enlargement of the pericardium and ventricle in larvae treated with Cd at a concentration of 10 μM. Barjhoux and collaborators reported the same result in Japanese madeka exposed to Cd. In their study, a progressive decrease in HR was observed after 7 days post-fertilization (dpf) [78].

Little interest is given to the cardiotoxic effects of Hg in fish despite its important role in affecting human hearts. A few examples are given to highlight the cardiotoxic effects of Hg on fish. Monteiro et al. (2017) studied the effects of Hg on matrinxa (*Brycon amazonicus*) and Tahria (*Hoplias malabaricus*) at concentrations of 0.1 and 0.45 mg L^-1^ respectively [80]. In both species, the authors noted the alteration of myocardial development, cardiac function, and reduction of the relative contribution of ion channels like Ca^2+^. Hg also impaired the electrical conduction across the heart. In Tahria, Hg exposure induced bradycardia and altered the hemodynamics of both atrium and ventricles by increasing the duration of the ventricular AP and delaying the depolarization of the atrium and ventricle. Very recently, Um et al. (2022) studied the occurrence of mercuric sulfide (HgS) to induce cardiotoxicity in zebrafish larvae [81]. The result displayed that exposure to 30 mg HgS mL^-1^ over a period of 3 days could disrupt the normal function of the heart, which was significantly attenuated through the measurement of HR and cardiac output. Despite all these studies highlighting the cardiotoxic effects of HMs in fish, it is still unclear how to translate fish-based cardiotoxic results to humans but very recently Maciag and collaborators developed a protocol for testing pharmacological drugs (doxorubicin, adrenaline, and terfenadine) inducing cardiotoxicity. The authors demonstrated that larvae of zebrafish showed the basic symptoms of cardiotoxicity, which mimic the human response hence supporting the use of zebrafish as efficient model to study CVDs [82].

### 3.2 Neurotoxic impact

Neurotoxic effects of many stressors may lead to behavioral and neuromotor changes affecting fish perception. HMs are able to affect the whole brain starting from transcriptional [83] to behavioral levels [84], [85].

In this line, it was proved that metals including Cd and Hg can cross the Blood Brain Barrier (BBB) and induce damages to the brain. In our previous work, we followed the expression of ABC transporters in peacock blennies exposed to a sublethal concentration of CdCl_2_ (2 mg L^-1^). Our results displayed that Cd significantly upregulated the expression of *abcc2* which is involved in GSH transport and Cd efflux as complexes with GSH. Downregulation of *ache* mRNA and AChE enzyme activities were also noted suggesting that Cd directly deactivate the enzyme catalytic site or removes cofactors from their sites [86]. Jebali and collaborators followed the exposure of the greater amberjack *Seriola dumerilli* to Cd and noted the inhibition of AChE activities after fish exposures to 100 and 250 μg Kg^-1^ over a period of 48 h [87]. Exposure of peacock blennies to 66 μg HgCl_2_ L^-1^ increased the expression of *abcb1*, which encodes a transmembrane transporter P-glycoprotein targeted to pump xenobiotics from cells. Hg was also an effective inhibitor of both *abcc1* and *abcc2* mRNA genes sharing the same function as *abcb1* [83].

Coordination and visual perception with body movements are closely limited to the optic tectum (OT). Many studies focused on the effects of trace metals on the eye and olfactory epithelium [16], [83], [88], [89], [90], [91], [92]. Exposure of adult and larvae zebrafish to Cd at different concentrations showed multiple disorders including an attenuated olfactory-based avoidance response to predator cues. In 10 μM CdCl_2_ exposed larvae, a clear decrease in fish body length, brain size, and eye size was recorded [91]. In the OT of *Salaria pavo*, histological sections of samples exposed to CdCl_2_ displayed the prevalence of circulatory disturbances including blood vessel abnormalities, congestion, and spongiosis after 4 and 15 days of exposure [16]. At day 10, fish suffered from regressive changes including atrophy of OT, vacuolization, fusion of layers and detachment, necrosis, and decrease of granular and mononuclear cells. As described by Mishra and Devi (2014), vacuolization and neuronal degeneration lead to organ atrophy [93]. Likewise, the breaking of the ventricular layer may lead to the enlargement of the adjacent ventricle or to the inflammation of the cerebrospinal fluid compartment [94].

For Hg, the main accumulation site in the OT is the periventricular layer (C1). Exposure of *Salaria pavo* to Hg over a period of 15 days revealed that alterations of the regressive pattern were very pronounced showing vacuolization, necrosis, atrophy, and fusion of layers [83]. In their study, Pereira and collaborators noted the loss of cells in the OT of the feral fish *Liza aurata* treated with moderate concentrations of Hg [92].

The cerebellum plays a key role in the circadian rhythm. Exposure of fish to Cd can cause blood vessel abnormalities, congestion, spongiosis, dilatation of Purkinje cells, clumping and hypertrophy of mononuclear cells, atrophy, vacuolization, and layer detachments. Changes related to circulatory disturbances could be the cause of *abc* transporter gene inhibitions as hypothesized in our previous work [16]. Al-Bairuty et al. (2013) found necrosis in the histological sections of the cerebellum [94]. The authors noted that blood vessel abnormalities in the ventral surface of the molecular layer contributed to the loss of locomotor circadian activity. Also, decreased Purkinje cells may influence the physiological activity of organisms [95]. Exposed fish to low doses of MeHg may delay fish apprenticeship while high doses may induce loss of apprenticeship in affected fish [96]. Exposure of peacock blennies cerebellum to 66 μg Hg L^-1^ induced circulatory disturbances, regressive, and progressive changes. The most pronounced alterations were congestion and a decrease in granular and Purkinje cells. Uema et al. (2001) [89] related the loss of Purkinje cells to hypoxia while Kaoud et al. (2011) [97] associated congestion of blood capillaries with encephalitis. In fish, loss of behavioral equilibrium is closely linked to alterations of the sensory organs [98]. All pathological changes occurring in the cerebellum may affect the capacity of fish to extract information from its environment thus, facilitating an appropriate decision-making process.

### 3.3 Section III: common signal pathways of Cd and Hg affecting heart and brain tissues

Many signal pathways are adopted by HMs to affect the different compartments of the body [99], [100], [101]. The most common cardiotoxic and neurotoxic effects of Cd and Hg are through oxidative stress and voltage-gated channels. In this section, we will describe how Cd and Hg may affect heart and brain tissues through the generation of ROS, induction of lipid peroxidation (LPO), and change in the activities of enzymes involved in oxidative defense. We will also detail how Cd and Hg can affect voltage-gated channels hence altering the normal function of both heart and brain tissues.

### 3.4 Oxidative stress

Metal that does not exhibit a redox potential may disrupt antioxidant defenses [102]. HMs have this propriety; they are able to induce oxidative stress by generating reactive oxygen species (ROS) [103]. It was well documented that an increment of ROS and an imbalance in antioxidant enzyme activities increase the risk factor of developing CVDs [104], [105]. In mammals, the production of ROS and generation of LPO induce apoptosis and lead to neurodegenerative diseases [83], [106], [107].

**Fig. 2:**
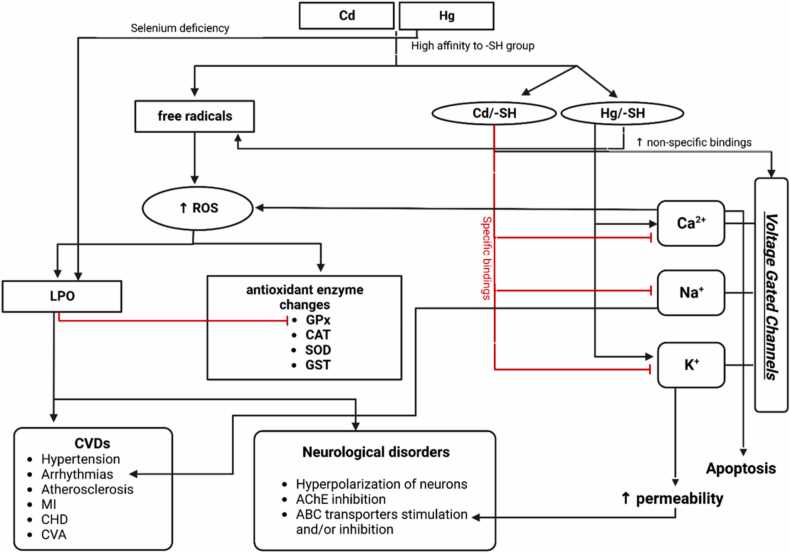
Cd and Hg-mediated common signaling pathways. Cd and Hg are able to produce free radicals, hence increase the production of ROS leading to LPO and changes in antioxidant enzyme activities including GPx, CAT, SOD, and GST. Hg induces LPO via selenium deficiency and blocs the production of GPx. LPO is one of major causes leading to CVDs (hypertension, arrhythmias, atherosclerosis, MI, CHD, CVA) and neurological disorders (hyperpolarization of neurons, AChE inhibition, ABC transporters stimulation and/or inhibition, ….). Cd and Hg directly affect the function of AChE which may be involved in neurobehavioral disorders.

Cd and Hg produce ROS and generate oxidative stress through Fenton reactions leading to a decreased number of copper and iron ions involved in the response to oxidative stress [108]. Once complexed to a cysteine-rich protein like metallothionein (MT), Cd concentration may increase by 3,000-fold times [109] because when the metal binds to MTs, its substitutes for zinc and copper in metalloenzymes and increases its affinity to sulfhydryl (-SH) groups [110]. It was reported that in Cd-affected hearts, baseline HRs increased when Cd induced the production of ROS thus, affecting the mitochondrial function (Fig. 2) [79]. Exposure of 6 dpf Japanese medaka *Oryzias latipes* to increased concentrations of Cd showed an increase in HRs followed by a progressive drop in HRs at 7 dpf. With 10 μM in Cd-treated larvae, authors noted the enlargement of both pericardium and ventricle resulting in a BP drop [79].

In Hg-exposed organisms, oxidative stress is the earlier biological response leading to LPO [111]. After absorption, Hg forms complexes with protein cysteine residues and thus decreases cellular antioxidant levels in cells. It was shown that HgCl_2_ dramatically affected electron transport and oxidative phosphorylation by generating oxidative stress [112]. As a result, ROS generation may lead to the development of CVDs including arrhythmias, hypertension, and atherosclerosis plaque development [113], [114]. Similar to Cd, Hg is a direct catalyst in Fenton reactions [115], [116], [117], [118]. To induce CVDs, Hg adopts different signal pathways (Fig. 2). Hg may induce LPO via selenium deficiency which is a necessary co-factor for glutathione peroxidase (GPx) activity that acts against hydrogen peroxides [119]. The result is thus promoting LPO which may lead to atherosclerosis, increase the risk of MI, congenital heart defects (CHD), and CVA [58], [104], [105], [120], [121], [122], [123]. Likewise, one of the indirect effects of MeHg in ROS enhancement is through metal-binding to –SH groups which inactivate antioxidant thiol compounds or peroxide-scavenging enzymes including GPx [124] thus reducing both membrane and plasma antioxidant enzyme activities.

### 3.5 Voltage-gated channels

When Cd and Hg bind to –SH groups, three different types of voltage-gated channels (Ca^2+^, Na^+^, and K^+^) are affected [125]. The mechanism by which Cd alters these ion channels is well proposed. For example, the non-specific binding of Cd on the surface negative charges of the plasma membrane increases is a possibility [126]. Another possibility of Cd-affecting ion channels is the binding of Cd to each channel in a specific manner. As a result, the bloc of the ion-selective pore of the channel may affect the opening and closing of channel gates as suggested by Johnson et al. [127].

In the cardiac tissue, the long plateau phase of AP is maintained by the balance between Ca^2+^ influx and K^+^ efflux [128]. In the presence of Cd, depression of the early plateau may occur [129]. Cd exposure can be also associated with the disruption of Na ions after acute toxicity [130]. The metal is a putative inhibitor of INa: at different concentrations, Cd was able to inhibit INa in rainbow trout [131]. In the presence of Hg, disruption of Ca^2+^ homeostasis results in cell apoptosis and death. It is also supposed that organic Hg increases the intracellular levels of Ca^2+^ by stimulating the influx of extracellular Ca^2+^ and immobilizing intracellular stores. In this line, MeHg significantly increased free Ca^2+^ ion levels in nerve cells, and disrupt Ca^2+^ homeostasis leading to oxidative stress [132]. Hg has also the capacity to affect cardiac Na^+^ homeostasis by promoting the oxidation of cysteinyl residues and by bridging adjacent –SH groups and consequently blocking Na^+^ channels [133]. This disruption facilitates the development of arrhythmias as suggested by Kuruta et al. [134]. In CNS, when binding to –SH groups in tubulin, MeHg inhibited the organization of microtubules, essential for CNS development [135], [136]. This binding promotes the blockade of Ca^2+^ channels in neurons [137]. It was also demonstrated that Hg and its inorganic form increased the permeability of chloride channels which is linked to the hyperpolarization of neurons [138].

### 3.6 Section IV: assessment of cardiac toxicity and neurotoxicity of Cd and Hg using zebrafish

It was largely described that fish is the best understood aquatic organism considered an excellent bioindicator of environmental changes, especially water pollution [139]. Among fish species, zebrafish is the most commonly used model with many advantages which make it an excellent tool in environmental toxicology including its optical transparency, ex-utero development, rapid nervous system development, lower cost (compared to in vivo rodent models), and adaptability for high throughout the screening. The genome of zebrafish was fully sequenced and expressed more than 70% of homology with the human genome [140]. Also, amino acid sequences of functionally relevant proteins are highly conserved in Zebrafish [141], [142]. It was demonstrated that zebrafish possessed orthologues for 86% of human drug targets [143] which is why the fish is now being well used to discover novel pathways and toxicity mechanisms to investigate human diseases [144]. Easy to manipulate its genome, zebrafish is now giving answers for multiple unknown roles of genes related to human diseases. Human and zebrafish cardiac muscles exhibit almost similar anatomical structures and physiological functions with slight anatomical differences as mentioned above. Both muscle cells share similar sarcomere and actin filament structures; they are both mitochondria-rich [145]. The electrical activities regulating the cardiac rhythm of the heart as well as the genes involved in repolarization by K^+^ channel function are orthologues [146]. Zebrafish have been proven to successfully identify with more than 85% rating of excellence cardiovascular toxins [147].

During the last 3 decades, zebrafish have been used to study the developmental neurotoxicity of multiple chemicals. Numerous studies displayed high similarities in the neurodevelopmental system between zebrafish and mammals. The comparative basic structure of CNS between zebrafish and mammals showed that both species shared all major domains as well as the same neurotransmitters such as GABA, glutamate, dopamine, serotonin, histamine, and AChE [148]. Like mammals, fish showed changes in neurodevelopmental sensitivity to hair-cell death caused by aminoglycoside antibiotics, the neomycin [149]. Another example is the morpholino knockdown of the *pink1* gene involved in Parkinson’s human disease [150], [151]. In the previous sections, we showed that metals are able to affect both heart and brain tissues. Based on multiple assessment processes, Cd and Hg were proved to be putative agents of cardio and neurotoxicity. Below we explain the most utilized techniques to assess the effects of HM exposures to zebrafish. In Table 1, we will summarize the most studied assessment assays.

**Table 1:** Most studied assessment assays to evaluate cardiotoxic and neurotoxic effects of Cd and Hg.

	***CARDIOTOXICITY***			***NEUROTOXICITY***		
**s**	*Lines*	*Target studies*	*References*	*Lines*	*Target studies*	*References*
**Transgenic line**	myl7:GFP; gata:DsRed	Defect in cardiac muscles	[152]	mitfa w2/w2	Visual assays	[153]
	Cmcl2	Erythrocytes	[154]	Pou4f3 and atoh7	Superficial interneurons	[155]
	gCaMP	Heart specific calcium sensors	[156]	Tg (mpvf:eGFP; npvf:C1V1-mCherry; npvf:GCaMP6s-p2A-TagRFP)	NPVF neuron circuits in the hypothalamus	[157]
	Fli1. EGFP	Vasculature/blood vessel	[158]	Tg (crh:RFP and otpa3kb:GCaMP3.0)	CRH neurons	[159]
	-			Tg (nkx2.2a:mEGFP)	Monitor of neurotoxins	[160]
**Oxidative stress**	LPO; antioxidant enzymes		[161]	LPO		[162]
**Tissue function**	Echocardiography		[163]	AChE activity		[16], [83]
	HR		[164]	visual responses through GECI		[165]
	SV			ERK		[166]
	EF			-		
	CO					
**Tissue structure**	Histology; Micro-CT		[167]	Histology, IHC, GALA4-UAS		[168]
**Gene expression**	-		*-*	1. *ache* 2. oxidative stress (*cat*, *gpx*, *sod*) 3. inflammatory reaction (*cox2*, *no*, *pge2*, *tnfα*) 4. detoxification (*abc*, *mt*, *tap*) 5. apoptosis (*bax*, *c-jun*) 6. DNA repair (*gadd*, *rad5*) 7. Mitochondrial process (*box1*)		[169]
**Behavioral assays**	-		-	Early embryonic movements		[170]
				Anxiety-like behavior		[169]
				Aggression		[171]
				Behavior-related vision (phototaxis, optomotor sand optokinetic responses, rheotaxis, antipredator behavior)		[85], [155], [172], [173], [174], [175], [176], [177], [178]

### 3.7 Exposure of zebrafish (embryos/adults) to HMs

Experimental designs conducted on zebrafish differ between embryos and adults. Briefly, adult zebrafish must be maintained in standard conditions in terms of water parameters including photoperiod. Target concentrations of HMs are supplied in the water of each experimental tank and during the experiment, the water must be changed every 2 days to guarantee the concentration of the HM treatment [179]. For zebrafish embryos, there are different ways to dissolve the metal right before starting the experiment. The most common HM dissolution is in zebrafish embryo media also called “egg water”. The egg water is mostly composed of NaCl, KCl, MgSO_4_, H_2_O, and Ca(NO_3_)_2_ and the value of each component slightly varies from one study to another.

### 3.8 Cardiotoxicity assessment

**Use of transgenic fish**: The concept of using transgenic fish has been well documented. The technique allows specific monitoring without the need for sophisticated instruments. Transgenic zebrafish for environmental toxicology in response to HMs was well established [180]. For example, the Zfin line was well used to reveal defects in cardiac muscle, erythrocytes, heart-specific calcium sensors, and vasculature/blood vessels using myl7:GFP, gata:DsRed, cmcl2, gCaMP and fli1.EGFP respectively [152], [154], [156], [158] (Table 1).

**Antioxidant enzyme activities**: Oxidative stress is an indicator of cardiovascular abnormalities in the most studied mechanisms of chemical inducing cardiotoxicity [181]. Antioxidant enzymes such as GPx, CAT, GST, and SOD are tested at both the transcriptional level through the expression of related genes and the physiological level through the measurement of protein activities. For example, the deletion of GSH triggers the disorders of the redox balance [161]. Decreased activities of both GPx and SOD activities accompanied with a down-regulation of the enzymes at the transcriptional level is a kind of acclimation to metal toxicity [15]. High expression of GPx and SOD at the basal levels might contribute to the protection of cells against metals [16] (Table 1).

**Measurement of Cardiac function parameters**: Doppler echocardiography is the most used technique in the assessment of cardiac functions in adult zebrafish. **Benslimane et al. (2019)** adapted a mouse Doppler echocardiography platform to measure cardiac flow velocities in adult zebrafish [163]. In their study, the authors placed and oriented the fish in a way that the probe is perpendicular to the fish to assess blood flow velocities at the AV canal. For the OFT valve, the angle of the probe was adjusted to 45° with the fish horizontal axes. For the head, another probe is placed toward the cranial end and the base toward the caudal end. All these probe orientations gave aligned ultrasound signals and blood flow direction.

HRs, Stroke Volume (SV), Ejection Fraction (EF), and Cardiac Output (CO) represent other parameters to assess the cardiac function. Altogether, allow the estimation of the functional state of the heart chambers. Imaging of a beating heart and flowing blood can be done on transparent embryos. Images of a beating heart are taken at two precise time points when the ventricle is totally contracted (end systole-ES) or fully relaxed (end diastole-ED) [164].

In embryos at 72-hpf, hearts are imaged using the same magnification and orientation as for the adults. A stereo-microscope is used to visualize the effects of the treatments on the cardiac structure and function [182]. High-speed time-lapse movies of the heart and tail in 1000 frames per 10 s at 100X magnification are recorded to measure the heartbeat and the blood flow in the tail. Two major blood vessels are analyzed: the Posterior Cardinal Vein (PCV) and the Dorsal Aorta (DA) to determine the effect of the treatment on four cardiac parameters: heartbeat, blood flow velocity, vessel diameter, and shear stress.

By using an algorithm from ViewPoint the heart beats per minute can be measured to calculate other functional cardiac parameters such as cardiac output. Also, the ventricular volumes and myocardial thickness can be measured to assess the presence of cardiomyopathy [163], [183] (Table 1).

**Heart structure:** To answer the question if exposure to HMs affects the heart of exposed fish, analysis of the heart macrostructure development is well recommended [167]. At the end of treatments with chemicals, whole zebrafish embryos or fresh hearts of adult fish are fixed in 4% PFA. After dehydration and embedding in paraffin wax, sagittal sections of heart samples are stained and observed under a microscope. Micro-CT is a more detailed technique that enables the generation of 3D heart volumes with high-resolution visualization of the perfused heart [184]. In this technique, microfilm cast are created for control and exposed fish by perfusing microfilm into the cardiac cavity and the surrounding vascular limens using glass capillary micro-needles [185]. For imaging, zebrafish embryos are loaded into X-Ray transparent capillaries and imaged using a microcomputed tomography scanner. The 3-D geometries are used to quantify ventricular chambers, volume, and AV valve orifice sizes (Table 1).

### 3.9 Neurotoxicity assessment

**Use of transgenic fish**: As for the assessment of cardiotoxicity, transgenic zebrafish are generated for specific purposes related to the neurological system [168], [186], [155], [159], [157], [187]. In transgenic zebrafish, the most used lines are:

1. mitfa^w2/w2^ [153], Tg (elav3:GCaMP5G)^2^ [188], Tg (foxD3:GFP)^zf104^ [189], [190], Tg (flh:EGFP)^U711^ [190], Tg (lhx2a:Gap43YFP)zf177 [191] for both visual and olfactory assays.
2. Tg (npvf:eGFP), Tg (npvf:C1V1-mCherry), Tg (npvf:GCaMP6s-p2A-TagRFP) to study NPVF neurons circuit in the hypothalamus [157].
3. Pou4f3:GFP and atoh7 used to study superficial interneurons in tectal Neuropil [155].
4. Tg (crh:RFP) and Tg (otpa3kb:GCaMP3.0) to analyze CRH neurons in response to stressor intensities [159].
5. Tg (nkx2.2a:mEGFP) is used as a highly sensitive monitor tool for neurotoxins [160] (Table 1).

The assessment of the neurotoxic effects of HMs in zebrafish can be divided into the following:

√ Behavioral assays,
√ Brain structures,
√ Antioxidant enzyme activities,
√ Gene expressions,
√ Brain function,

**Behavioral assays**: neurotoxic effects in most cases of intoxication can lead to changes in fish behavior (zebrafish larvae and adults). The multiple assays on fish behavior are described as fellow:

• *Early embryonic movements*: It is very easy to assess the movements of embryos in the early developmental stages since zebrafish embryos can show first movements from 17 hpf. Fish motility can be performed using an automated video tracking system that records free swimming in individual fish larvae and adult zebrafish. Through tracking systems, multiple parameters can be calculated such as speed, distance, pattern, and time (McGrath and Li, 2008).
• *Anxiety-like behavior*: Thigmotaxis, freezing, and erratic swimming are the most common sensitive parameters measuring anxiety-like behavior [192], [193], [194]. Novel tanks and light/dark preference tests are also used in anxiety-like behavior assessments.
• *Aggression*: Zebrafish, as a schooling species, defends its territory. Aggressive behaviors consist of alternating and/or coincident fin displays and attack behaviors while displays are represented by the erection of dorsal, pectoral, and anal fins. The slapping of the caudal fin is also among the displays. Aggressive fish showed biting motions and directed swimming at the attacker. To assess aggression, an inclined mirror is used.
• *Behavior-related vision*: Impairment of the olfactory system has a strong impact on fish behavior and survival. The specific endpoints of fish vision include predator avoidance, prey capture, optomotor, and optokinetic responses [155], [172]. Different assays are considered to assess visually-guided behaviors.

1. Phototaxis: light/dark transition test is showed to assess impact of neuroactive compounds [173], [174]. Because zebrafish are active during the day, they prefer to spend more time in light than in dark [195]. During the assessment, larvae are placed in a two-compartment chamber with both conditions (dark and light). Fish that tend to move toward the illuminated chamber are considered to be intact in terms of functional vision. The absence of such a reaction indicates an impairment in vision [175].
2. Optomotor and optokinetic responses: The optomotor technique is based on double-cone photoreceptors (red/green). Later, Avdesh et al. (2012) tested the preference of zebrafish for 4 colors: red, yellow, green, and blue [176]. The authors demonstrated that fish has an equal preference for red, green, and yellow colors and less preference for blue. The test of optomotor response is based on the reflection of fish. Animals reflexively keep pace with a grated drum below the swimming chamber. Larvae with altered vision remain in place while fish with intact vision is supposed to swim along the grating and end up at one end of the chamber. The optokinetic technique consists of a smooth pursuit movement that stabilizes the image on the retina of fish, then a fast resetting saccade is done to repeat another cycle. In an arena where fish are placed, a jelly-like substance is added to prevent larvae from swimming. An appropriate software allowed the detection of eye movements [84].
3. Rheotaxix: Rheotaxis is the behavior by which fish orient themselves and swim against the current. Sensory hair cells of the neuromast are able to transduce pressure changes in the surrounding medium into neuronal signals. These pressures enhance the opening of ion channels and enable the detection of acoustic stimuli and hydrodynamic flow. In zebrafish, the mechanosensory hair cells are similar to those of humans [85]. In the rheotaxis test, behavior, orientation, and swimming performance can be assessed in zebrafish larvae from 5 dpf. Altered behavioral responses and hair-cell death are the main effects of HMs observed in zebrafish [196], [197].
4. Antipredator behavior: The assay, having a direct relation with the visual system consists of presenting fish with a large shape modeled after a predator or predatory fish. As a result, fast start response increases. Endpoints of the assay include time to initiate an attack, number of attacked prey, capture efficiency, and time to cessation [177], [178] (Table 1).

**Brain structure assessment**: Behavioral measurements alone cannot be used as indicators of neurotoxicity because such impairments are not specific to neurotoxic chemicals. Confirmative assays must take place to improve behavioral responses in fish.

• Immunohistochemistry (IHC) is a more performed technique than histology in larvae tests since the technique is applied on whole-mount animals because of their small body size and transparency without losing much time in preparing sample sections. Another more performant structure assessment is of using transgenic fish, which offers a real-time assessment of the neurogenesis [168]. Another technique most commonly used on zebrafish is the binary system of GAL4-UAS (Table 1).

**Antioxidant enzyme activities:** As mentioned in section III, exposure to Cd and Hg may lead to oxidative stress via the increased production of ROS and LPO. Antioxidant enzymes are also considered in oxidative stress assays generated by Cd and Hg. Among these enzymes, we noted CAT, SOD, GPx, GST, and GSH activities [162], [198] (Table 1).

**Assessment of gene expressions at the transcriptional level**: The interaction of toxicants with molecules starts early at the transcriptional level. Repression of the expression of genes involved in the neurological function will inhibit the formation of the appropriate protein, hence the alteration of the brain function. Multiple genes involved in the response to stressors are investigated including AChE and ABC transporters (ABCB, ABCC, ABCG) which are involved in mechanisms of transport, metal pump, and detoxification of chemical compounds. Abnormal expression of these genes may reflect the powerful neurotoxic effects of chemicals [16], [83]. In some works, antioxidant enzyme activity assays are followed by the study of their expression at the transcriptional level. Among these enzymes, relative expressions of *sod* and *cat* are the most studied [162], [198], [199], [200], [201]. On the other side, the relative expression of genes involved in the inflammatory reaction of the brain is well studied. Brain inflammation can be assessed following the expression of inflammatory markers like *cox2*, *no*, *pge2*, and *tnfα* [201]. Gonzalea and collaborators compared the expressions of many gene biomarkers in the brain of zebrafish and revealed that *gadd* and *rad5* (DNA repair), *mt2* and *tap* (detoxification process), *bax* and *c-jun* (apoptosis), *box1* (mitochondrial process), and *mnsod* (oxidative stress) are specific genes for both Cd and Hg exposures [199] (Table 1).

**Brain function assessment:** The best-known biomarker in neurotoxicity is AChE [16], [83], [202]. Studying the AChE activity can be followed by the expression of *ache* mRNA at the transcriptional level [203] in the brain of exposed organisms. Different described microscope techniques including the confocal and epifluorescence microscopies enable imaging of brain activities of zebrafish [188], [204], [205], [206], [207]. Brain imaging allows the identification of changes in neuronal activity even if they are small changes. These identified changes may disturb the behavior of fish and affect their survival. To analyze the visual responses of zebrafish larvae, Niell and Smith (2005) injected a calcium indicator dye into the tectal neuropil [165]. The genetically encoded calcium indicators are called “GECIs”. As a result, nerve generation with calcium sensors is considered to measure the activity of the whole brain. Another technique that can be used is based on the endogenous sensor phosphorylated ERK [166]. Brain Cd imaging has been used to identify chemoconvulsant-induced changes in different regions of the brain [208] (Table 1).

## 4 Conclusion

In the present review, we concluded that Cd and g exert toxic effects on human health. Smoking cigarettes is the main source of intoxication with Cd that may increase the risk of CVDs including hypertension, PAD, and CHD. In many human cases of intoxication, it was demonstrated that Hg causes CVDs diseases and neurological disturbances. Toxicological assays on fish models confirmed these findings and through research studies, common signal pathways of Cd and Hg causing heart and brain tissue disturbances displayed that oxidative stress and imbalance in the voltage-gated channels are the main sources of cardiotoxicity and neurotoxicity. Many biomarkers were developed to better assess the toxicity of Cd and Hg and most of these biomarkers are studied in zebrafish that is now being used to discover pathways and toxicity mechanisms of many chemicals, drugs, and other environmental pollutants.

## Declaration of Competing Interest

The authors declare that they have no known competing financial interests or personal relationships that could have appeared to influence the work reported in this paper.

## Data Availability

No data was used for the research described in the article.
